# Low back pain and patient-reported QOL outcomes in patients with adolescent idiopathic scoliosis without corrective surgery

**DOI:** 10.1186/s40064-015-1189-y

**Published:** 2015-08-07

**Authors:** Takahiro Makino, Takashi Kaito, Masafumi Kashii, Motoki Iwasaki, Hideki Yoshikawa

**Affiliations:** Department of Orthopaedic Surgery, Graduate School of Medicine, Osaka University, 2-2 Yamadaoka, Suita, Osaka 565-0871 Japan; Department of Orthopaedic Surgery, Osaka Rosai Hospital, 1179-3 Nagasone, Kita, Sakai, Osaka 591-8025 Japan

**Keywords:** Adolescent idiopathic scoliosis, Low back pain, Prevalence, SRS-22r, JOABPEQ

## Abstract

**Purpose:**

To reveal the prevalence of low back pain (LBP) and association between LBP and patient-reported QOL outcomes (JOABPEQ and SRS-22r) in patients with adolescent idiopathic scoliosis (AIS) without corrective surgery.

**Methods:**

Ninety-eight female patients with AIS without corrective surgery who answered JOABPEQ, SRS-22r, and VAS for LBP were included. The scores of all subdomains in JOABPEQ and SRS-22r were calculated. From the standing radiographs, we measured the Risser grade and radiographic parameters regarding the curve magnitude, coronal and sagittal balance, and spinopelvic alignment. Furthermore, we recorded whether the patients were undergoing brace treatment at the time of visiting our outpatient clinic. The patients with VAS >30 mm (moderate or severe LBP) were designated as Group P; VAS ≤30 mm, Group N. All variables were compared between the groups.

**Results:**

The prevalence of LBP (VAS >0 mm) was 34.7% and that of moderate or severe LBP was 16.2%. All subdomain scores in JOABPEQ and those for *function* and *pain* in SRS-22r were significantly smaller in Group P than Group N. The subdomain scores for *self*-*image* and *satisfaction/dissatisfaction with management* in SRS-22r did not differ between the groups. The age, Risser grade, radiographic parameters, and whether the patients were undergoing brace treatment did not differ between the groups.

**Conclusions:**

The prevalence of LBP was 34.7%, which was approximately three times higher than that previously reported in Japanese pupils without scoliosis. The patients with LBP demonstrated poorer QOL outcomes associated with LBP regardless of radiographic parameters, patients’ self-image and satisfaction with treatment.

## Background

Adolescent idiopathic scoliosis (AIS) is not a rare spinal condition, and its prevalence is approximately 1–3% (Weinstein et al. 2008; Ueno et al. 2011). As severe curve progression can affect not only the cosmetic appearance and trunk balance but also the respiratory and cardiac function (Weinstein et al. 2008), most reports associated with AIS have mainly focused either on the prevention of curve progression or surgical techniques and their outcomes.

In contrast, little attention has been paid to back pain and the related quality of life (QOL) in patients with AIS without surgeries, partly because AIS has been considered to be painless (Weinstein et al. 1981; Ramirez et al. 1997). However, several studies have indicated that the prevalence of back pain is significantly higher in patients with AIS than in control populations without AIS (Mayo et al. 1994; Sato et al. 2011). Furthermore, the difference in radiographic parameters or patient backgrounds between patients with AIS who experience pain and those who do not remains unclear.

A number of trends in patient evaluation have recently resulted in the development and growing use of patient-reported outcome measurements designed for assessing the functional status and health-related QOL (HRQOL). The Japanese Orthopaedic Association Back Pain Evaluation Questionnaire (JOABPEQ) is a recent patient-reported evaluation questionnaire, drafted from the Short Form-36 and Roland–Morris Disability Questionnaires, which assess QOL associated with low back pain (LBP) (Fukui et al. 2009). In the field of scoliosis, the Scoliosis Research Society (SRS)-22r questionnaire has been widely used; the Japanese SRS-22r has been previously validated (Hashimoto et al. 2007). Many studies have focused on QOL outcomes of patients with AIS after corrective surgery or on the difference in QOL outcomes between the patients with and without AIS. However, the difference in QOL outcomes between the patients with AIS with and without LBP remains unclear.

For an effective patient care, it is essential to know the distinguishing characteristics between “painful” and “painless” patients with AIS. The purposes of the present study were (1) to identify the prevalence of LBP and association between LBP and patient-reported QOL outcomes (JOABPEQ and SRS-22r) in patients with AIS without surgery and (2) to identify the predictors for LBP, including radiographic and psychological assessments, in patients with AIS without surgery.

## Methods

The present study was a retrospective review of prospectively collected data. It was approved by the Research Ethics Committee of Osaka University Hospital (No. 11360).

Among the consecutive 111 female patients with AIS (Cobb angle >10°) without corrective surgery who visited our outpatient clinic between July 2013 and June 2014, 98 patients who answered JOABPEQ, SRS-22r, and visual analog scale (VAS) identifying the worst LBP during the previous week (range 0–100 mm) were included in this study. The mean age was 14.7 years (range 10–18). According to the previous reports, patients with VAS ≤30 mm were defined as having no pain or mild pain; VAS >30 mm, moderate or severe pain (Collins et al. 1997; Kelly 2001). The patients with VAS values of LBP >30 mm were designated as Group P (patients with moderate or severe LBP), and those with VAS values of LBP ≤30 mm were designated as Group N (patients without LBP or with mild LBP).

### QOL assessments

All patients answered the JOABPEQ and SRS-22r. We calculated the score of each subdomain of JOABPEQ (*low back pain*, *lumbar function*, *walking ability*, *social life function*, and *mental health*) and SRS-22r (*function*, *pain*, *self*-*image*, *mental health*, and *satisfaction/dissatisfaction with management*), according to the provided formulae.

### Radiographic assessments

From the full-length standing spinal posteroanterior and lateral radiographs of all patients, the following parameters were digitally measured by the first author (TM) blindly to the outcomes (VAS, JOABPEQ and SRS-22r) on a flat-panel monitor at our hospital using a built-in imaging software (Centricity WebDX: GE Healthcare Japan, Tokyo, Japan) with a precision of 1° in angles and 0.1 mm in distances:

1. Posteroanterior radiograph
  Risser grade, Cobb angles of the main thoracic (MT) and thoracolumbar/lumbar curves (TL/L), location (thoracic or thoracolumbar/lumbar) of major curve, apical vertebral translation of MT and TL/L, T1 tilt, L4 tilt, and the distance between the C7 plumb line and central sacral vertical line (C7-CSVL).
2. Lateral radiograph
  Thoracic kyphosis, lumbar lordosis, pelvic incidence, pelvic tilt, and the distance between C7 plumb line and posterosuperior corner of S1 (sagittal vertical axis: SVA).

### Treatment data

We recorded whether the patients were undergoing brace treatment at the time of visiting our outpatient clinic.

### Statistical analysis

Statistical analysis was performed using IBM SPSS Statistics Version 22 (IBM, Armonk, NY, USA). For univariate analysis, the Mann–Whitney U test was used to compare all the subdomain scores of JOABPEQ and SRS-22r, age, Risser grade, Cobb angles (MT, TL/L, major curve), apical vertebral translation of MT and TL/L, T1 tilt, L4 tilt, C7-CSVL, thoracic kyphosis, lumbar lordosis, pelvic incidence, pelvic tilt, and SVA between Groups P and N. The Fisher’s exact probability test was used to compare location of major curve and whether the patients were undergoing brace treatment. Multivariate logistic regression analysis (stepwise) was performed to detect the demographic or radiographic risk factors for VAS values of LBP >30 mm. For the multivariate logistic regression analysis, the variables showing values of *p* < 0.25 in univariate analysis were used after confirming no correlation (|ρ| < 0.8 in Spearman’s rank correlation) with each variable. Differences were considered statistically significant at *p* < 0.05.

## Results

**Fig. 1:**
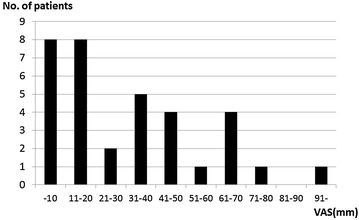
Frequency distribution chart of visual analogue scale (VAS) for low back pain in the adolescent idiopathic scoliosis patients. The median VAS value was 27.5 mm (range 4–100 mm).

The prevalence of LBP (VAS >0 mm) was 34.7% (34/98 patients) and that of moderate or severe LBP (VAS >30 mm) was 16.2% (16/98 patients). The median VAS value of the patients with LBP was 27.5 mm (range 4–100 mm) (Fig. 1). Group P comprised 16 patients with the mean age of 15.4 years (range 11–18); Group N, 82 patients with the mean age of 14.6 years (range 10–18).

Table 1 shows the subdomain results for the JOABPEQ and SRS-22. All of the JOABPEQ subdomain scores were significantly smaller in Group P than in Group N (*low back pain*, 49.9 vs. 95.8, *p* < 0.001; *lumbar function*, 87.4 vs. 98.1, *p* < 0.001; *walking ability*, 95.6 vs. 99.1, *p* = 0.022; *social life function*, 76.3 vs. 97.5, *p* < 0.001; *mental health*, 68.6 vs. 77.8, *p* = 0.021). Moreover, the SRS-22r subdomain scores for *function* and *pain* were significantly smaller in Group P than in Group N (*function*, 4.4 vs. 4.8, *p* < 0.001; *pain*, 3.8 vs. 4.7, *p* < 0.001). The subdomain scores for *self*-*image* and *satisfaction/dissatisfaction with management* in the SRS-22r were not different between the groups.

**Table 1:** Subdomain scores of the Japanese Orthopaedic Association Back Pain Evaluation Questionnaire (JOABPEQ) and Scoliosis Research Society-22r Questionnaire (SRS-22r)

	Group P (VAS >30 mm, n = 16)	Group N (VAS ≤30 mm, n = 82)	*p*
JOABPEQ			
Low back pain	49.9 ± 27.2	95.8 ± 11.1	<0.001
Lumbar function	87.4 ± 17.9	98.1 ± 7.3	<0.001
Walking ability	95.6 ± 11.2	99.1 ± 5.3	0.022
Social life function	76.3 ± 17.5	97.5 ± 8.3	<0.001
Mental health	68.6 ± 14.0	77.8 ± 16.5	0.021
SRS-22r			
Function	4.4 ± 0.5	4.8 ± 0.3	<0.001
Pain	3.8 ± 0.5	4.7 ± 0.4	<0.001
Self-image	2.8 ± 0.6	3.0 ± 0.5	0.182
Mental health	4.0 ± 0.7	4.3 ± 0.7	0.053
Satisfaction/dissatisfaction with management	3.2 ± 0.5	3.4 ± 0.7	0.152

Values are expressed as means ± standard deviations.

Mann–Whitney’s U test.

Table 2 shows the results for demographic data, radiographic parameters, and treatment data. The age, Risser grade, and all of the radiographic parameters showed no difference between the groups. Only 12.5% of patients in Group P were undergoing brace treatment; 25.6% of patients in Group N were undergoing brace treatment, though the difference between the groups was not significant. The age (*p* = 0.190), Risser grade (*p* = 0.168), Cobb angles in major curve (*p* = 0.145), apical vertebral translation of MT (*p* = 0.180) and C7-CSVL (*p* = 0.244) were identified as the potential risk factors for moderate or severe LBP in the univariate analysis; however, the multivariate analysis demonstrated none of them was risk factor for it.

**Table 2:** Demographic, radiographic, and clinical data

	Group P (VAS >30 mm, n = 16)	Group N (VAS ≤30 mm, n = 82)	*p*
Age	15.4 ± 2.3	14.6 ± 2.0	0.190*
Risser grade (0–5)	3.9 ± 1.2	3.5 ± 1.4	0.168*
Cobb angle (°)			
Main thoracic	36.1 ± 11.4	32.0 ± 14.3	0.343*
Thoracolumbar/lumbar	32.1 ± 14.8	27.5 ± 9.0	0.381*
Major curve	40.9 ± 12.8	35.5 ± 11.8	0.145*
Location of major curve (thoracic:thoracolumbar/lumbar)	12:4	52:30	0.567**
Apical vertebral translation (mm)			
Main thoracic	28.2 ± 15.5	22.7 ± 16.5	0.180*
Thoracolumbar/lumbar	18.8 ± 14.7	18.2 ± 13.2	0.939*
T1 tilt (°)	3.4 ± 3.4	3.7 ± 3.8	0.826*
L4 tilt (°)	12.1 ± 8.3	10.2 ± 5.6	0.596*
C7-CSVL (mm)	15.3 ± 9.7	12.8 ± 8.9	0.244*
Thoracic kyphosis (°)	22.0 ± 10.6	21.0 ± 11.6	0.600*
Lumbar lordosis (°)	−50.8 ± 16.9	−49.6 ± 10.7	0.471*
Pelvic incidence (°)	43.3 ± 11.0	43.2 ± 9.1	0.965*
Pelvic tilt (°)	11.9 ± 8.4	10.6 ± 8.0	0.623*
SVA (mm)	19.1 ± 11.0	19.8 ± 16.9	0.900*
Brace treatment (y:n)	2:14	21:61	0.345**

Values are expressed as the means ± standard deviations.

C7-CSVL indicates the distance between the C7 plumb line and central sacral vertical line.

SVA indicates the distance between the C7 plumb line and posterosuperior corner of S1.

* Mann–Whitney’s U test.

** Fisher’s exact probability test.

## Discussion

This study revealed that the prevalence of LBP was 34.7% (34/98 patients) in patients with AIS without surgery. Furthermore, 16.2% of these patients (16/98 patients) suffered from moderate or severe LBP. In patient-reported QOL assessments, LBP in AIS was associated with a decrease in QOL related to pain, lumbar function, walking ability, social life function, and mental health, but not associated with self-image and satisfaction/dissatisfaction with the management. The location of the major curve, radiographic parameters for the curve magnitude, coronal and sagittal balance, and spinopelvic alignment were not associated with LBP.

In contrast to adult scoliosis, AIS has been recognized as a spinal deformity without pain (Aebi 2005). Ramirez et al. (1997) have speculated that the prevalence of back pain in AIS was similar to that in the general pediatric and adolescent population; however, this was not a comparative study. Mayo et al. (1994) have reported in their comparative retrospective large cohort study (n = 2,092) that the patients with AIS experienced back pain more than the control subjects (current back pain, 44 vs. 24%). Further, Sato et al. (2011) have reported an epidemiological study of 43,630 Japanese pupils (age 9–15 years) who were screened at school for scoliosis, identifying 51 students with AIS and 32,083 students without it and the prevalence of back pain in patients with AIS was markedly higher than that found in patients without scoliosis (point prevalence, 27.5 vs. 11.4%). Although the definition of back pain (intensity, location, or duration of pain) and patient backgrounds (curve magnitude, sex, social background, or race) were different across these studies, the prevalence of LBP in the patients with AIS in our study was similar to that previously report from Japan (Sato et al. 2011) and was approximately three times higher than that reported in Japanese cohorts without scoliosis.

In the field of spinal scoliosis, SRS outcomes have been widely used for patient-reported outcome measurements. Rushton and Grevitt (2013) have recently reviewed the studies using SRS outcomes for evaluating HRQOL in patients with AIS without treatments; in their review, the subdomain scores for *pain* and *self*-*image* were significantly lower in patients with AIS than in those without scoliosis. However, few studies focused on the QOL difference between patients with AIS with and without LBP. Moreover, the SRS outcomes are not specifically LBP-related QOL outcome measurements and cannot detect what types of LBP-related disability affect the patients with AIS. The advantage of the JOABPEQ is that it allows an independent evaluation of five subdomains of LBP-related disability (*low back pain*, *lumbar function*, *walking ability*, *social life function*, and *mental health*). The JOABPEQ results in our study revealed that not only physical functions such as lumbar function, walking ability and social life function but also mental conditions deteriorated in patients with LBP.

Several predictors for back pain in patients with AIS have been previously observed (Weinstein et al. 1981; Ramirez et al. 1997; Petcharaporn et al. 2007; Smorgick et al. 2013; Cochran and Nachemson 1985). The association between back pain and radiographic parameters remains controversial (Weinstein et al. 1981; Ramirez et al. 1997; Petcharaporn et al. 2007; Smorgick et al. 2013). Our study has revealed that none of the radiographic parameters, including the curve magnitude, coronal and sagittal balance, and spinopelvic alignment, differed between the patients with AIS with moderate or severe LBP and with no or mild LBP. Furthermore, the multivariate logistic regression analysis failed to demonstrate the risk factor for moderate or severe LBP in AIS. The present study was limited as we could not evaluate the curve flexibility. Smorgick et al. (2013) have reported that patients with a rigid lumbar curve suffered more from back pain. The magnitude of curve was relatively mild and global balance maintained in both coronal and sagittal plane in most cases in this study, and so the difference in radiographic parameters could not be apparent.

LBP in patients with AIS cannot be explained only by radiographic parameters. It has been recently argued that psychological factors play a significant role not only in chronic pain but also in acute pain, even in adolescents (Linton 2000; Korovessis et al. 2010). We hypothesized that an inferior self-image or dissatisfaction with the management could cause LBP in patients with AIS. In this present study, the subscale scores for *mental health* in JOABPEQ were significantly smaller in the patients with moderate or severe LBP than those with no or mild LBP; however, the related subscale scores of SRS-22r (*self*-*image* and *satisfaction/dissatisfaction with management*) revealed no significant difference between the patients with moderate or severe LBP and with no or mild LBP. The subdomain score for *self*-*image* in SRS-22 and -24 decreased only when the Cobb angles exceeded 40°–50° (Watanabe et al. 2005a, b; Parent et al. 2010). In our study, the mean Cobb angles were approximately 30° in the MT and TL/L curve; therefore, no difference was observed in *self*-*image* scores in SRS-22r.

Another limitation of our study was that the location of LBP was not clearly defined because no information about the location of pain was included in the design of either JOABPEQ or SRS-22r. Several authors have suggested that back pain in patients with AIS occurred more frequently in the concavity of the curve (Weinstein et al. 1981), rib hump (Weinstein et al. 1981), inter- and supra-scapular lesion (Weinstein et al. 1981; Dickson et al. 1990), thoracolumbar region (Dickson et al. 1990), or upper and middle right back (Sato et al. 2011). Further studies are required for investigating the association between the location of LBP and patient-reported QOL outcomes.

In conclusion, we investigated the prevalence of LBP and related patient-reported QOL outcomes in patients with AIS without surgery. The prevalence of LBP was 34.7% and that of moderate or severe LBP was 16.2%. In patient-reported QOL assessments, LBP in patients with AIS was associated with a decrease in QOL correlated with pain, lumbar function, walking ability, social life function, and mental health. Though not only the radiographic parameters for the curve magnitude, coronal and sagittal balance, and spinopelvic alignment but also patients’ self-image and satisfaction with treatment assessed by patient-reported QOL outcome measurements failed to correlate with LBP in patients with AIS, we should keep in mind that LBP in patients with AIS is not rare condition and can cause deterioration of patients’ QOL.
